# Metabolite Fingerprinting Based on ^1^H-NMR Spectroscopy and Liquid Chromatography for the Authentication of Herbal Products

**DOI:** 10.3390/molecules27041198

**Published:** 2022-02-10

**Authors:** Florentinus Dika Octa Riswanto, Anjar Windarsih, Endang Lukitaningsih, Mohamad Rafi, Nurrulhidayah A. Fadzilah, Abdul Rohman

**Affiliations:** 1 Center of Excellence, Institute for Halal Industry and Systems, Universitas Gadjah Mada, Yogyakarta 55281, Indonesia; dikaocta@usd.ac.id (F.D.O.R.); anjarwindarsih2@gmail.com (A.W.); 2 Division of Pharmaceutical Analysis and Medicinal Chemistry, Faculty of Pharmacy, Campus III Paingan, Universitas Sanata Dharma, Maguwoharjo, Sleman, Yogyakarta 55282, Indonesia; 3 Research Division for Natural Product Technology, National Research and Innovation Agency (BRIN), Yogyakarta 55861, Indonesia; 4 Department of Pharmaceutical Chemistry, Faculty of Pharmacy, Universitas Gadjah Mada, Yogyakarta 55281, Indonesia; lukitaningsih_end@ugm.ac.id; 5 Department of Chemistry, Faculty of Mathematics and Natural Sciences, Kampus IPB Dramaga, IPB University, Bogor 16680, Indonesia; mra@apps.ipb.ac.id; 6 International Institute for Halal Research and Training (INHART), International Islamic University of Malaysia (IIUM), Gombak 53100, Malaysia; nurrulhidayah@iium.edu.my

**Keywords:** herbal medicine, herbal authenticity, spectroscopic, chromatographic, chemometrics

## Abstract

Herbal medicines (HMs) are regarded as one of the traditional medicines in health care to prevent and treat some diseases. Some herbal components such as turmeric and ginger are used as HMs, therefore the identification and confirmation of herbal use are very necessary. In addition, the adulteration practice, mainly motivated to gain economical profits, may occur by substituting the high price of HMs with lower-priced ones or by addition of certain chemical constituents known as *Bahan Kimia Obat* (chemical drug ingredients) in Indonesia. Some analytical methods based on spectroscopic and chromatographic methods are developed for the authenticity and confirmation of the HMs used. Some approaches are explored during HMs authentication including single-component analysis, fingerprinting profiles, and metabolomics studies. The absence of reference standards for certain chemical markers has led to exploring the fingerprinting approach as a tool for the authentication of HMs. During fingerprinting-based spectroscopic and chromatographic methods, the data obtained were big, therefore the use of chemometrics is a must. This review highlights the application of fingerprinting profiles using variables of spectral and chromatogram data for authentication in HMs. Indeed, some chemometrics techniques, mainly pattern recognition either unsupervised or supervised, were applied for this purpose.

## 1. Introduction

Herbal medicines (HMs), which remedies and medication using herbal (botanical) components, are known as one of the most traditional forms of health care. For over hundreds of years, HMs have been widely used for the prevention and treatment of certain diseases. The World Health Organization (WHO) estimates that about 65–80% of the world’s population use herbal medicines, particularly in developing countries with limited access to modern medication. Certain botanicals have been widely used as herbal components in traditional medicines in some societies, such as *Curcuma longa* L. (turmeric) and *Curcuma xanthorrhiza* Lam (Java turmeric) [1]. HMs may contain single or multiple herbs in a single drug preparation; this is why herbal medicines could have a complex mixture of many herbs that contain many chemical compounds [2]. According to the WHO guidelines, HMs are herbs (including crude materials which can be derived from lichen, fungi, algae, and parts of higher plant materials, either entirely or powdered), herbal materials (including fresh juices, gums, fixed oils, essential oils, resins, and dry powders of herbs), herbal preparations, and finished herbal products. In recent years, the increased awareness and public concern on issues related to the adulteration practice and the authenticity of HMs has attracted scientists to develop the analytical methods for the authentication analysis of HMs, which are complex in nature and have variability in their chemical contents [3,4].

The activity of HMs depends on the chemical compositions present in herbs, which may vary depending on the plant species, age, harvesting season, location of growth, drying processes, and some other environmental factors. As a consequence, in order to ensure the reliability and repeatability of pharmacological and clinical research studies, and to guarantee the consistency of the final product quality, the quantitative analysis of all bioactive components (phytochemicals) in HMs is required. However, determination of all phytochemicals is time-consuming, laborious, and unsuitable for confirming the synergist effects among HMs. Therefore, it is very important to apply the analytical techniques based on identifying all metabolites represented by metabolite fingerprinting for the authentication and geographical origin of plant species [5].

Metabolite fingerprinting can be referred to as the analysis of as many components as possible in HMs within a system, including their detection and the subsequent data treatment of the obtained results using some chemometrics techniques for differentiation or classification of the evaluated samples. In metabolite fingerprinting, the identification and quantification of the detected metabolites may not be a necessity [6]. Several methods have been reported for the authentication of HMs through fingerprint profiling including spectroscopic-based methods such as FTIR spectroscopy [7], DNA fingerprinting-based authentication [8], as well as DNA using Next-Generation Sequencing (NGS) [9]. In addition, there are some chromatographic methods including high-performance thin-layer chromatography [10] and ultra-performance liquid chromatography (UPLC) using some detectors such as the diode array detector (DAD) [11] and mass spectrometers [12]. Reviews on the application of certain techniques for the authentication of herbal medicines were also reported, such as near-infrared and mid-infrared spectroscopy in combination with chemometrics [13], chromatography combined with chemometrics [14], and DNA barcoding [15].

Herbal medicine is most widely found in complementary and alternative medicine therapies used throughout the world [16]. A herb is the part or whole of plants used for medicinal and therapeutic applications. Herbal medicines typically consist of plants or plant extracts containing some active constituents, which frequently work synergistically [17]. Chemical constituents with some medicinal benefits are referred to as active ingredients or active principles, such as the curcuminoid present in the *Curcuma* species. The presence and levels of active components depend on several factors including plant species, time and season of harvesting, soil types, and other environmental conditions [18]. Currently, over 80% of the world population uses herbal medicines as preventive and promotive agents either in developing or developed countries [19]. As a consequence, the increased use of herbal products has also led to some actions of adulteration and abuse of herbal products, leading to consumers and producers’ disappointment, and in some instances the abuse and adulteration can cause health problems [20].

The discrimination and authenticity of herbal products are emerging issues [21], especially in countries which develop alternative medicines as primary health care, such as China, India, Germany, and Indonesia [22]. Herbal authentication is mainly related to improper labeling and economic adulteration. Motivated by economic profits, high-quality herbal medicines may be adulterated with lower-quality herbs, thus having a less expensive price to defraud the consumer. In the other case, in Indonesia, the misuse addition of *Bahan Kimia Obat* (chemical drug ingredients) may increase the potential toxicological risk in the routine consumption of HMs [23]. The adulteration practice also involved the substitution, either in part or the whole of expensive herbal components, with cheaper and inferior herbal products. An authentic herbal product can be defined as herbal when it complies with the description or labelling provided by the producers, which includes plants’ composition, its geographic region of origin, and the variety or species of ingredients [24].

Han et al. [25] reported the adulteration practices in Chinese herbal medicines. Using the DNA barcode database of Traditional Chinese Medicine (TCM), 1436 samples representing 295 medicinal species from seven primary TCM markets in China have been investigated. Of the 1260 samples, approximately 4.2% of herbal medicines were identified as adulterated. Some herbal components such as Ginseng Radix et Rhizoma, Radix Rubi Parvifolii, Dalbergiae odoriferae Lignum, Acori Tatarinowii Rhizoma, Inulae Flos, Lonicerae Japonicae Flos, Acanthopanacis Cortex, and Bupleuri Radix are among the targets of adulteration. The survey also reported that adulterants were present in the Chinese market. In order to assure the quality of labelled herbal medicines, it is essential to establish the methods to identify its authenticity either by checking the composition of the herbal ingredients or monitoring batch-to-batch reproducibility [26].

The authentication of HMS using fingerprinting techniques is not inevitably focused on the identification of all metabolites present in the evaluated samples but rather on the pattern recognition, which indicates the classes or certain classes of compounds in the used analytical conditions. This technique also provides a qualitative analysis allowing for the reliable identification and authentication of HMs even if the levels of the characteristic chemical components are not exactly the same for the evaluated HMs samples [27]. Another study reported the opportunity to develop a rapid detection test to identify contaminants in botanicals extracts [28]. Fortunately, the use of some chemometrics techniques applied for spectroscopic and chromatographic data, especially pattern recognition methods, could highlight differences and similarities among evaluated samples [14].

## 2. Methods

This review article was written by identifying, investigating, and assembling several review articles, original articles, books, and relevant sources on metabolite fingerprints from reputable worldwide databases including Web of Science, Scopus, and PubMed. The literature searching was carried out between September and December 2021. The keywords explored during the literature investigation were “metabolite fingerprinting”, “^1^H-NHMR”, “liquid chromatography”, “chemometrics”, and “authentication”.

## 3. Chemometrics

**Figure 1:**
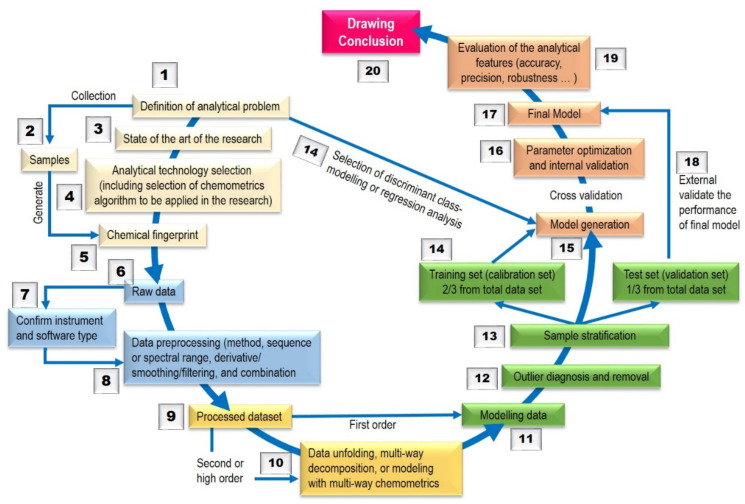
General analytical flow of chemometrics modelling. Adapted from [5].

The fingerprint profiling of HMs using LC–MS/MS and ^1^H-NMR is basically the chemical profiling of some common chemical components of biologically active and/or chemical characteristics. This profiling should be featured by the fundamental attributions of the similarity and differences of chemical components present in HMs, such as the profiling of authentic and adulterated HMs using LC–MS/MS and ^1^H-NMR [29]. Using chromatographic and spectroscopic fingerprint profiling, the authentication and identification of HMs can be accurately carried out even if the concentration of the chemically characteristic constituents are not exactly the same for different samples of this HM. However, in any HMs, there are hundreds of unknown components and many of them are in low amounts, which results in high amounts of instrumental (chemical) responses which are difficult to be handled. Fortunately, the statistical techniques known as chemometrics (Figure 1) are powerful enough to treat big chemical data [30].

Chemometrics can be described as the employment of mathematics and statistics techniques to analyze several chemical data [31,32]. In HMs’ authentication, chemometrics were classified according their purposes and techniques, such as exploratory data analysis, exploratory data analysis, unsupervised pattern recognition, and supervised pattern recognition. The most widely used chemometrics method for the purpose of exploratory data analysis is principal component analysis (PCA). Unsupervised pattern recognition algorithms commonly used in HMs’ authentication are similarity analysis (SA) and hierarchical clustering analysis (HCA), whereas the supervised pattern recognition algorithms commonly applied in the field are soft independent modeling of class analogy (SIMCA), linear discriminant analysis (LDA), partial least-squares–discriminant analysis (PLS–DA), k-nearest neighbors (KNN), and least-squares–support vector machine (LS–SVM) [33].

PCA, the most popular chemometrics technique in chemometrics, was widely applied for the exploratory data analysis. General features of the PCA as an unsupervised pattern recognition are its ability to reduce the dimensionality of the datasets as well as its increasing interpretability but at the particular time minimizing information loss [34]. The idea behind PCA was the generation of principle components (PCs) as an orthogonal linear transformation considering the variance occurring due to the variables of the data [35]. The PCA algorithm combined with chromatographic and spectroscopic techniques in HMs’ authentication was successfully applied for identifying the botanical raw material of *Panax notoginseng* [36]. Aided by the PCA technique, the ^1^H-NMR spectroscopy can be applied for analyzing 46 authentic rice samples according to their types [37].

SA, one of the unsupervised pattern recognition techniques, can be applied in HMs’ authentication by calculating the correlation coefficient and congruence coefficient. When the correlation coefficient and congruence coefficient result in a value of 1, it can be stated that two chromatograms are similar [38]. Other unsupervised pattern recognition techniques, namely HCA, were usually generated to find the highest similarity within clusters as well as the highest dissimilarity between clusters.

Supervised pattern recognition in HMs’ authentication was commonly implemented in generating the classification model according to experimental data by assigning unknown samples to a previously labelled sample class according to the pattern properties of chemical measurements [39]. LDA can be applied for maximizing the classification distance between several classes of HMs samples [40]. In the case of ‘ill conditioned’, when the number of samples are less than the number of the measured variables, as commonly found in chemistry data, the PLS–DA may improve the classification capability of the generated predictive model [41]. Other classification techniques such as SIMCA and KNN can be carried out for authenticating HMs in combination with spectroscopy methods [42]. By employing the PCA techniques for each class, the SIMCA classification was able to be built for producing a predictive model using only the significant components [43]. The KNN algorithm assumes that similar samples are present near to each other since similar things exist in close proximity [44]. Supervised pattern recognition according to the theory of statistical learning using regression and classification techniques, namely LS–SVM, can be applied in the fingerprint profiling of HMs [45].

## 4. Methods for Authentication of Herbal Medicines Using Metabolite Fingerprinting

For the authentication of HM species, WHO, USFDA, and the European Medicines Agency (EMEA) have stated that the authenticity identification of HMs is one of the first assays that should be performed for ensuring the quality of HMs and for discriminating HMs from related species or adulterated HM samples [1]. Therefore, rapid and accurate analytical approaches are essentially required the authenticity of HMs and prevention of the adulteration practice. There are some strategies typically employed for the authentication of HMs, namely using the classical approach, which applies single-component analysis (SCA) with targeted compounds, and analysis based on the non-targeted approach using the fingerprint profiling and metabolomics approach [46].

In SCA, the authentication of HMs was carried out by identification and quantification of specific chemical markers used as an indication of certain HMs. For example, curcuminoid compounds can be employed for identification of the Curcuma species. The addition of Curcuma longa or turmeric with other HMs could decrease the levels of curcuminoids which could be exploited for identification of the adulteration practice [47]. SCA has some advantages including high sensitivity and selectivity, ease in instrument operation, and simple data analysis, while the main drawback of SCA typically is related to the analytical method validation typically involving extensive sample preparation and multiple analysis [48]. Chromatographic-based methods are ideal methods for the analysis of targeted compounds due to its capability of providing good separation of targeted analyte(s) in HMs [49].

Metabolite fingerprinting has emerged as a powerful alternative technique to classical analytical methods (SCA) for the authentication of herbal medicine. In fingerprint profiling, the assumption was made in which the raw analytical data includes chemical information which is sufficient to answer biological questions. This task is typically done using a dataset of herbal samples from various states such as in the differentiation between authentic and adulterated herbal medicines, as well as when distinguishing the origins of plant species. By applying various analytical techniques in combination with chemometrics, it is possible to reliably extract some variables from chemical responses, allowing for the discrimination between authentic and adulterated herbal samples [50].

Due to its capability to separate HMs into the corresponding components, the Food and Drug Administration and the European Medicines Agency recommend the use of chromatographic techniques as the most suitable tools for metabolites, as represented by peaks in chromatograms. As a consequence, in the last years, many chromatographic methods have been developed for the fingerprint profiling of different HMs samples. Among these, thin-layer chromatography (TLC), high-performance thin-layer chromatography (HPTLC), high-performance liquid chromatography (HPLC), electrophoresis, and gas chromatography (GC) equipped with several detectors were used [14].

**Figure 2:**
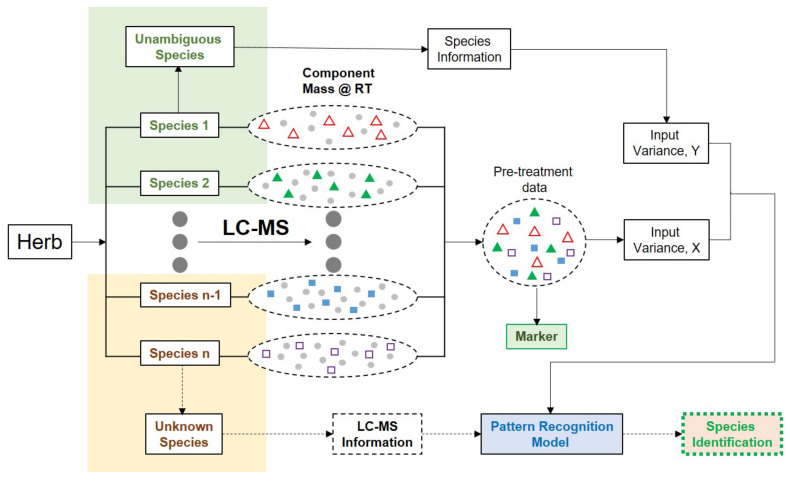
The simplified strategy illustrating the use of HPLC or LC–MS/MS for herbal identification intended for authentication analysis. X and Y represent the matrixes of input and output. RT represents retention time. Peak area and m/z are variables typically used during chemometrics analysis. Adapted from [31].

Chromatographic fingerprinting is an alternative method to distinguish authentic and adulterated HMs. Gao et al. used the comprehensive analysis for the authentication of HMS, namely (1) the full metabolic profiling of HMs by liquid chromatography or LC–MS/MS (2) global analysis of non-targeted compounds, employing the molecular feature extraction algorithm, (3) multi-variate statistical analysis for classification and prediction, and the characterization (4) marker compounds [30]. Gao et al. [31] proposed fingerprint profiling for herbal identification intended for authentication analysis (Figure 2). This strategy involved the algorithm for the automation of compound extraction in order to process the metabolic profiles of HMs at the level of individual molecular fragments without the assignment of the chemical structure. After that, the chemometrics analysis using variables of all chemical information was employed for obtaining the predictive model for the classification of HMs (authentic versus adulterated). The list of publications on HMs’ authentication is presented in Table 1.

### 4.1. Chemical Fingerprinting Using HPLC

Liquid chromatography has been successfully applied for the herbal standardization of Traditional Diabetes Herbal, which consists of plant materials reported to have anti-diabetic activity such as *Andrographis paniculata, Cinnamomum zeylanicum, Curcuma xanthorrhiza, Eugenia polyantha*, and *Orthosiphon stamineus* [52]. The HPLC fingerprint profile of UV detection at wavelengths of 210, 254, and 280 nm, as well as LC–MS have been successfully applied for the profiling of glucofarmaka antidiabetic jamu consisting of Sembung leaves (*Blumea balsamifera*), brotowali stems (*Tinospora crispa*), ginger rhizomes (*Zingiber officinale*), and pare leaves (*Momordica charantia*). LC–MS was applied for the identification of the chemical compounds present in the formula, such as 6- gingerol and 6-shogaol [49]. The combination of HPLC using a UV detector and UPLC with a photodiode array (PDA) detector was reported for the identification and authentication of raw materials used in HMs, namely *Panax notoginseng* (Burk.) F.H. Chen (known in China as Tianqi). The chemometrics of PCA, SIMCA, and PLS–DA were applied for the classification of rhizomes and roots of *P. notoginseng*. Some chemical markers were also identified, namely notoginsenoside R1, ginsenoside Rg1, ginsenoside Re, ginsenoside Rb1, and ginsenoside Rd [36].

*Andrographis paniculata* Nees (family Acanthaceae), one of the most promising herbal medicines in Southeast Asia and known as *Sambiloto* in Indonesia, was subjected to fingerprint profiling using HPLC–UV detection at 223 nm. The five samples of each *A. paniculata* genotype from 10 different origins in India were collected for HPLC analysis and the nine selected peaks were used as variables during the chemometrics analysis. PCA and SIMCA were applied for the classification of samples according to their origins. For the exploratory data analysis, PCA was used for searching the similarity among samples, while SIMCA was applied for the classification of the evaluated samples. The classification of *A. paniculata* from different origins was successfully carried out using SIMCA based on the predefined PCA model with correctness levels of 100% for all classes at the significance level of 0.05 [50].

Ultra-performance liquid chromatography with a diode array detector (UPLC-DAD) has been successfully reported for the authentication of *Rosa rugosa*, which is herbal used for traditional herbal medicine for treating diarrhea, stomachache, and menoxenia. Ten batches of *R. rugosa* were collected from different plantations in China to establish the fingerprint. UPLC fingerprint chromatograms of samples using 23 characteristic fingerprint peaks were verified for its similarity with those in professional analytical software as recommended by China’s compendia. The developed method was successfully applied for the differentiation of *R. rugosa* from different regions. Some biomarkers, namely gallic acid, ellagic acid, Kaempferol-3-*O*-sophoroside, hyperoside, and astragalin, were quantified using the validated method. The performance characteristics of the validated method meets the acceptance criteria [10].

### 4.2. Fingerprinting Using LC–MS/MS

Today, liquid chromatography–mass spectrometry tandem mass spectrometry (LC–MS/MS) has been reported for the analysis of bioactive components in herbal medicines either for quality control or for authentication analysis [11]. Using LC–MS/MS, the chemical structure characterization, molecular mass, information of fragmentation, retention time, and broad range of detection and high separation of analytical compounds can be achieved using this technique [51]. LC–MS/MS has been used for the identification of twenty markers for the authentication of traditional herbal formulas of Hyeonggaeyeongyo-tang (Chinese herbal medicines), consisting of 13 medicinal herbs including *Schizonepetae Spica, Forsythiae Fructus, Saposhnikoviae Radix, Angelicae*, etc. Some markers identified and determined using LC–MS/MS included gallic acid, geniposide, paeoniflorin, narirutin, etc. The presence of these markers along with its concentration could be used as a fingerprinting profile of this formula [52].

Rapid-resolution liquid chromatography combined with quadrupole time-of-flight mass spectrometry (RRLC–ESI/QTOF MS) was used for the fingerprint profiling of seven *Lonicera* species, namely *Lonicera japonica* (LJ), *L. confusa* (LC), *L. dasystyla* (LD), *L. fulvotomentosa* (LF), *L. hypoglauca* (LH), *L. macranthoides* (LM), and *L. similis* Hemsl. (LS). PCA-score plots revealed that seven groups according to the *Lonicera* species appear in clusters along three PCs. The samples of LJ, LF, and LM clustered clearly but several samples from LC, LD, LH, and LS were mixed with others. Therefore, the chemometrics classification using a support vector machines (SVM)-based classifier was used for classification and prediction. Using SVM, all samples were successfully classified into seven groups according to its species. The model was also accurately predictive of the uncontained batches of samples. By comparing with authentic compounds, some markers were identified, namely 7-epi-loganin (iridoid), lonicerin (flavonoid), macranthoidin B (saponin), macranthoidin A (saponin), luteolin (flavonoid), and agenin (saponin) [28]. These results indicated that the proposed SVM-based pattern recognition method could classify and predict *Lonicera* species.

### 4.3. Metabolite Fingerprinting Using ^1^H-NMR

Nuclear magnetic resonance (NMR) spectroscopy is a sophisticated molecular spectroscopy that has been extensively used for food and pharmaceutical product analysis, and it has been considered as a potential analytical method for the detection of non-halal substances [24]. The principle of NMR spectroscopy is based on the interaction of molecules with certain radio waves, resulting in the changes of spin direction and visually presented as several spectra NMR at different magnetic fields. NMR provides fingerprint spectra, which makes it useful for sample differentiation, including the detection of non-halal substances [25]. The instruments mostly used for the analysis operate at a frequency of 500–600 MHz. NMR is a versatile molecular spectroscopy technique because of its advantages, such as easy in-sample preparation, non-destructiveness, less-solvent requirements (considered as green analytical chemistry), less time required for analysis, high reproducibility and robustness, as well as the fact that it can be used for the analysis of heterogeneous samples simultaneously in the majority of cases [26]. The most common technique used is proton-NMR (^1^H-NMR) spectroscopy because it offers simplicity in the sample preparation, fast analysis, and it can be used even for crude extract analysis [27]. Other NMR techniques that play important roles in halal authentication analysis are carbon NMR (^13^C-NMR) and two-dimensional NMR (2D) techniques such as *J*-resolved, HSQC (heteronuclear single-quantum correlation), HMBC (heteronuclear multiple-bond correlation), and TOCSY (total correlation spectroscopy) [29,69]. The combined information from different NMR experiments enabled it to be employed in structural evaluations and for further elucidation processes [70,71]. 

*Curcuma longa* (Turmeric) and *Curcuma xanthorrhiza* (Java turmeric) are among the herbal components used in Indonesian traditional medicines. The main components in Curcuma species are curcuminoids. ^1^H-NMR spectroscopy-based metabolite fingerprinting in combination with multivariate analysis for the authentication *C. longa* from *C. heyneana* and *C. manga* was successfully applied [69]. The different types and levels of metabolites can be represented in the different chemical shifts in NMR spectra. In the regions of 0.00–3.00 ppm, *C. longa* has more signals with higher intensities, while *C. manga* revealed few signals with low intensities. *C. longa* also has more signals and higher intensities compared to *C. manga* in the regions of 5.50–10.00 ppm and no signals are observed after the region of 8.00 ppm. ^1^H-NMR metabolite fingerprinting in combination with PCA and OPLSA–DA using variables of the chemical shift was successfully used to classify the authentic powder of *C. longa* and that which adulterated with *C. heyneana* and *C. manga*. OPLSA–DA showed a good of fit (R^2^X = 0.912 and R^2^Y = 0.795) and good predictivity (Q^2^ = 0.711). OPLSA–DA successfully distinguished between pure and adulterated *C. longa* with C. manga even in 5% of adulterant concentration. Validation of OPLSA–DA by the permutation test showed that the OPLSA–DA model is reliable for authentication of *C. longa* [56]. Furthermore, the multivariate calibration of PLS was used to predict the adulterant levels in *C. longa*. The R^2^ value for the relationship between actual values of *C. heyneana* (*x*-axis) and predicted values (*y*-axis) based on responses in ^1^H-NMR was 0.9992 with the equation of y = 0.9992x + 0.0344, indicating that the model fits the data well. Meanwhile the PLS response plot for such a correlation yielded a R^2^ of 0.9982 with the equation of y = 0.9982x + 0.0745 for the prediction of *C. manga* levels as adulterants in *C. longa*. The RMSEC and RMSEP values obtained were relatively low, i.e., 0.94% and 0.83%, for *C. longa* adulterated with *C. heyneana*, as well as 1.37% and 1.34% for C. longa adulterated with C. manga. Low values of RMSEC and RMSEP indicated that the developed model revealed good accuracy and precision of the calibration models [57].

^1^H-NMR spectra in combination with the chemometrics of PCA and OPLS–DA (orthogonal projections to latent structures–discriminant analysis) was successfully applied for authentication of the *Curcuma xanthorrhiza* extract from *Curcuma aeruginosa*. During the adulteration of *C. xanthorrhiza* with *C. aeruginosa*, the levels of curcumin were decreased as the level of *C. aeruginosa* was increased, as determined by HPTLC. PCA using variables of ^1^H-NMR peaks was used for classification. Previously, ^1^H-NMR spectra were subjected to Pareto scaled to diminish the effects of variables that do not have important roles in classification using chemometrics of pattern recognition. The signals that do not have significant contribution on classification were removed. PCA using selected signals of ^1^H-NMR spectra clearly discriminated pure and adulterated *C. xanthorrhiza* with *C. aeruginosa*. Furthermore, OPLSA–DA successfully classified authentic *C. xanthorrhiza* and that which adulterated with *C. aeruginosa* resulted in acceptable statistical parameters with high R^2^X (0.965), R^2^Y (0.958), and Q^2^ (cum) (0.930). From these results, ^1^H-NMR spectra-based metabolite fingerprinting coupled with PCA and OPLS–DA offers a reliable method for assessing the adulteration of the adulteration practice and for evaluating the authentication of *C. xanthorrhiza* with *C. aeruginosa* [58]. A similar way was also applied for the authentication of *C. longa* with *C. heyneana*. The model using ^1^H-NMR spectra revealed a good fit and good predictivity as shown by the statistical values of R^2^X (0.85), R^2^Y (0.992), and Q^2^ (0.899). The results demonstrated that ^1^H NMR-based metabolite fingerprinting and chemometrics of multivariate analysis can be applied to distinguish pure and adulterated *C. longa* with *C. heyneana* [59].

Three chemometrics of PCA, PLSA–DA, and N-nearest neighbors (N3) based on the classical K-nearest neighbor using ^1^H-NMR spectra fingerprints were used as a novel strategy for the authentication method of *Polygoni multiflori Radix* (PMR), which is a widely used herbal medicine and functional food from *Cynanchi auriculati Radix* (CAR), the common adulterant to PMR. ^1^H-NMR spectra of PMR and CAR could be differentiated, especially at chemical shifts of 3.0–5.42. However, the ^1^H-NMR of PMR and CAR showed some similarities, therefore, in order to obtain the most significant chemical shifts and achieve rapid and intuitive analyses, the characteristic ^1^H NMR fingerprints were constructed. PCA, as an exploratory data analysis method, was used to describe the major classification based on the raw and characteristic ^1^H-NMR fingerprints. Using raw ^1^H-NMR data, the first two PCs accounted for 63.60% (PC1 = 50.49% and PC2 = 13.21%), and when using ^1^H-NMR fingerprints, the PC1 accounted for 60.23% and PC2 10.76%, contributing to 70.99% of what was obtained. Based on the PCs score plot, PMR and CAR samples revealed the separated clusters. N3 and PLS–DA using variables of characteristic ^1^H-NMR fingerprints could classify PMR and CAR with accuracy levels of 100% without any misclassification. From these results, ^1^H-NMR fingerprints in combination with PLS–DA and N3 could be an effective and reliable technique for the authentication of PMR from CAR [60]. 

The ^1^H-NMR spectroscopy-based metabolite fingerprinting approach combined with chemometrics has been used for the evaluation of Saffron (*Crocus sativus*) adulteration with saffron stamens, safflower, turmeric, and gardenia. Authentic and adulterated samples of Saffron were extracted using DMSO as the extraction solvent because DMSO could dissolves both hydrophilic and hydrophobic compounds. PCA using PC1 and PC2 could only differentiate Saffron samples adulterated with gardenia extracts and saffron adulterated with turmeric. Analysis using supervised pattern recognition of OPLS–DA employing one predictive and three orthogonal components provided R^2^X = 0.824, R^2^Y = 0.945, and Q^2^ = 0.923. Result showed that OPLS–DA successfully discriminates authentic Saffron from adulterated Saffron with four adulterants, as mentioned above. Investigation through an S-line plot revealed that metabolites of pirocrocin (1.12, 1.16, 2.08, 4.28, and 10.04 ppm) and crocins (1.96, 4.16, 5.40, 6.52, 6.64, 6.84, and 7.32 ppm) were found higher in authentic Saffron samples. Chemometrics of bidirectional PLS–DA, also known as O2PLS–DA, was also successfully used to detect the adulteration of Saffron. Performance using three predictive and three orthogonal components showed that all authentic Saffron samples clearly separated from all adulterated Saffron samples with R^2^X = 0.952, R^2^Y = 0.976, and Q^2^ = 0.960. These results suggested that this developed method resulted in reliable and reproducible results in assessing the authenticity of Saffron and determining the type of adulteration in Saffron at a minimum-level adulterant of 20% [61].

Determination of metabolite levels in *Capsicum annum* L. (serrano pepper) grown in two different origins has been performed using ^1^H-NMR metabolomics and chemometrics. ^1^H-NMR metabolomic profiling was carried out using a water extract of serrano pepper obtained from two areas, namely Veracruz and Oaxaca in Mexico. There were 40 metabolites identified from ^1^H-NMR spectra analysis and most of the metabolites possess nutritional functions such as glucose, fructose, sucrose (3.0 to 5.0 ppm), amino acids, carboxylic acids (0.5 to 3.0 ppm), and aromatic compounds (6.0–10.0 ppm). The ^1^H-NMR spectra from two different regions demonstrated a clear difference in the levels of glucose, fructose, sucrose, and citrate. The main difference between serrano pepper from Veracruz and Oaxaca is the presence of lactate in Oaxaca but no succinate, while there is a presence of succinate in Veracruz but no lactate. Analysis using PCA could differentiate serrano pepper from two different regions with R^2^ and Q^2^ values of 0.936 and 0.875, respectively. The supervised pattern recognition technique of OPLS–DA provided a better classification with R^2^X = 0.923, R^2^Y = 0.999, and Q^2^ = 0.996. Further investigation on the OPLS–DA loading plot found that metabolites of citrate, lactate, aspartate, leucine, and sucrose were the important metabolites for differentiation of serrano pepper from Veracruz, whereas metabolites of formate, malonate, fumarate, acetate, pyruvate, succinate, and phosphocholine were the important metabolites to differentiate serrano pepper from Oaxaca. These results suggested that ^1^H-NMR metabolomics and chemometrics could be used to determine metabolite compositions on serrano peppers grown in diverse areas [59].

Lee et al. used ^1^H-NMR-based metabolite fingerprinting combined with multivariate analysis to discriminate Asian red pepper powders distributed in Korea based on their geographical origins. Samples were obtained from three different regions, namely Korea (36 samples), China (17 samples), and Vietnam (nine samples). Samples were extracted by the sonication technique using mixtures of methanol-d4 and D_2_O (3:1 *v*/*v*) employing DSS (Sodium trimethylsilylpropanesulfonate) as an internal standard. ^1^H-NMR spectra analysis revealed some of the metabolites such as sucrose, α-glucose, β-glucose, unsaturated fatty acids, tryptophan, phenylalanine, alanine, tyrosine, adenosine, uridine, histidine, and kaempferol. Red peppers from Vietnam had a higher content in tyrosine and alanine compared to China and Korea. Metabolites of α-glucose, β-glucose, tryptophan, and adenosine were found in high amounts in red peppers from Korea than in China and Vietnam, whereas samples from China possessed lower kaempferol than Korea and Vietnam. Chemometrics using canonical discriminant analysis (CDA) could correctly classify 15 blind samples according to their geographical origins. One sample from China was misclassified as a Korean spice because of its high levels of α-glucose and β-glucose [63].

The metabolomics approach using ^1^H-NMR spectroscopy and chemometrics of pattern recognition has been successfully used for quality control as well as for the geographical origin discrimination of black pepper (*Pepper nigrum* L). CDCl_3_ and CD_3_OD were found to be the most suitable solvents for polar and non-polar compound extraction, respectively. The piperine signal was found to be dominant in the NMR spectra since piperine is known to be the major compound in black pepper. Extracted using CDCl_3_, piperine was observed at a chemical shift of 1.55–1.67 ppm (multiplet, m), 3.51–3.62 ppm (m), 5.96 ppm (singlet, s), 6.42 ppm (doublet, d, J = 14.6 Hz), 6.70–6.74 ppm (m), 6.76 ppm (d, J = 8.0Hz), 6.88 ppm (doublet of doublets, dd, J = 1.6, 8.0 Hz), 6.97 ppm (d, J = 1.6 Hz), and 7.38 ppm (doublet of doublet of doublets, ddd, J = 14.6 Hz). These results were in accordance with the signal of piperine in published literature measured using the ^1^H-NMR spectroscopy technique. PCA was used to differentiate piperine samples from Srilanka, Brazil, and Vietnam. Samples extracted using CDCl_3_ provided better group clustering than by that using CD_3_OD with overlapped clusters. Classification using OPLS–DA demonstrated better discrimination both in the CDCl_3_ and CD_3_OD extract. The OPLS–DA model using CDCl_3_ extract demonstrated a R^2^X of 0.914, R^2^Y of 0.956, and Q^2^ of 0.877. Meanwhile, the OPLS–DA model using CD_3_OD showed a better model than CDCl_3_ as demonstrated by its value of R^2^X (0.977), R^2^Y (0.962), and Q^2^ (0.928). Identification of potential metabolite markers for discriminating black pepper from different origins using the Box-Cox plot found that piperine, fatty acids, and isoleucine were the important metabolites for sample discrimination [64].

The combination of ^1^H-NMR spectroscopy and chemometrics of PCA and PLSA–DA has been used for the differentiation of celery (*Apium graveolens* L. var. dulce) from different geographical origins, namely China, Taiwan, and Australia. All celery samples showed high intensities in the chemical shift of 3.00–5.00 ppm which correspond to the carbohydrate compounds and mannitol found as the primary metabolites. Further analysis provided a number of metabolite classes in a celery, such as carbohydrates, amino acids, phenolic acids, and organic acids. Untargeted metabolomics using NMR spectroscopy data and PCA could differentiate both the leaf and stem of celery extract from three different origins. The samples from Taiwan were separated from Australia and Taiwan, and the metabolites important for differentiation were mannitol (3.66–3.78 ppm), which is higher in Taiwan; citric acid (2.5 ppm); and 4-hydroxybenzoic acid (6.94–7.78 ppm) which is found to be lower. Meanwhile, the differentiation between celery samples from Australia and China was due to higher sucrose (4.18) and lower glutamine (2.46 ppm). Differentiation analysis using PLS–DA obtained better discrimination than using PCA because OPLS–DA could maximize the covariance among variables through its orthogonal variables. Some of the metabolites that played important roles for discrimination were mannitol, asparagine, sucrose, malic acid, and glutamine, which is in accordance with the important metabolites found using PCA. All samples were correctly classified without misclassification, indicating good performance of the OPLS–DA model [65].

Discrimination of *Mentha* species grown in different origins of Algeria has been determined using NMR-based metabolomics and chemometrics. NMR spectra showed that the signals of rosmarinic acid were dominant in three *Mentha* species. Rosmarinic acid could be identified by the presence of doublet proton signals at 7.49 ppm and 6.29 ppm with a similar coupling constant (15.9 Hz). These signals corresponded to the protons of the double-bond of conjugated protons bonded with the ester group of rosmarinic acid. Three *Mentha* species (*Mentha pulegium* L.*, Mentha* × *rotundifolia* (L.) Huds., and *Mentha spicata* L) were divided into two groups using PCA. Analysis using OPLS–DA provided a better discrimination result than in PCA. All three *Mentha* species were successfully classified into three different classes with good predictive ability (Q2 = 0.978). An investigation on variables’ potential for sample discrimination found that signals of 3.65 to 4.17 ppm, which correspond to carbohydrates, and signals of 5.38 to 7.67 ppm, which belong to aromatic derivatives, are potential biomarkers to distinguish *Mentha pulegium* from the other two species [72]. Meanwhile, the potential discrimination biomarker of *Mentha rotundifolia* and *Mentha spicata* obtained from S-plot analysis was terpenoid (1.24 to 2.93 ppm). Moreover, as observed through a loading scatter plot, flavonoid glycosides were found to be potential discriminating biomarkers [66].

Farag et al. used NMR spectroscopy and the chemometrics approach to discriminate two cinnamon species, namely *Cinanmomum verum* and *Cinnamomum cassia*, for authentication purposes. Cinnamon is made from the bark of cinnamon species, which has been widely used as a spice in the world. *Cinnamomum verum*, also known as Ceylon cinnamon, has golden color, delicate flavor, and is known to have some health benefits such as antidiabetic, antifungal, and anti-allergy activity. Meanwhile, *Cinnamomum cassia*, known as Chinese cinnamon, has a red–brown color and contains a higher amount of coumarin. Ceylon cinnamon has a higher price than *Cinnamomum cassia* and is often adulterated with *Cinnamomum cassia* for economic purposes. Therefore, an analytical method capable of cinnamon authentication is highly required. Sample extraction was carried out using methanol-d4 and HMDS (hexamethyldisiloxane) as the internal standards. NMR measurement was performed using an NMR spectrometer operating at a frequency of 599.83 MHz. NMR spectra analysis found some flavor compounds such as eugenol, cinnamic acid, glycerol, cinnamaldehyde, cinnamaldehyde dimethyl acetal, acetic acid, and *o*-hydroxycinnamaldehide. Chemometrics analysis using PCA and OPLS–DA was used to discriminate two species of cinnamon. Result showed that both PCA and OPLS–DA could be used for differentiating Ceylon cinnamon and Chinese cinnamon. The compound of eugenol was found to be a potential biomarker of Ceylon cinnamon, whereas the presence of fatty acids could be used as a potential biomarker of Chinese cinnamon [67].

The application of NMR-based metabolomics and chemometrics has been used for the chemical fingerprinting of three edible *Allium cepa* L. (onion) obtained from Italia. Three onions, namely white onion, red onion, and yellow onion, were determined as their metabolomes. Samples were extracted using D_2_O to extract polar compounds and deuterated chloroform for non-polar extracts. NMR spectra acquisition was performed using a 600 MHz NMR spectrometer both for one-dimensional (1D) and two-dimensional (2D) NMR. Some metabolites were identified, such as aromatic carbohydrates, amino acids, organic acids, and organosulfur. Metabolites of carbohydrates were dominant in white onion (about 95%) and the presence of fructo-oligosaccharides (FOS) was unique in white onion because it was absent in red and yellow onions. For amino acid compositions, yellow onion has the highest concentration of amino acids, followed by red onion and white onion. Furthermore, the lack of pyruvate and α-hydroxybutyrate was found in white onion. Chemometrics analysis using unsupervised PCA and supervised PLS–DA was performed to classify onion samples based on their metabolic compounds. Results showed that PCA and OPLS–DA successfully discriminated three different cultivars of onion. Glucose, sucrose, FOS, and sterols were the important metabolites to differentiate white onion. The important metabolites in red onion were sterols and glucose, whereas the metabolites that play important roles for the differentiation of yellow onion were methiin, free isoalliin, γ-glutamyl-isoalliin, glutamine, malate, and choline [68].

Metabolite fingerprinting in HMs studies was widely applied in detecting biological features by chemometrics techniques. However, there are still limitations and challenges remaining for further study. The reliability of recovering metabolites from plant extracts remains the biggest drawback in the field of metabolite fingerprinting. Another study reported that the application of ^1^H-NMR remained as a disadvantage of the signals in the NMR spectrum that are rather small, with extensive overlap in several regions of the spectrum [73]. In the future, an in-depth understanding of metabolites systems, pathways, and their interactions should be reported to strengthen the scientific information from HMs studies [74].

## 5. Conclusions

The use of herbal medicines (HMs) as complementary and alternative medicine is becoming popular in the general population worldwide. Parallel to the increased trends of the application of HMs as alternative therapies either for preventive or promotive treatments, some research activities dealing with the quality control, standardization, and authentication of HMs also increased. The efficacy of HMs depends on their quality and authenticity. Fingerprint profiling based on spectroscopy, especially ^1^H-NMR and chromatographic techniques hyphenated with mass spectrometers (LC–MS/MS), in combination with classification chemometrics has emerged as a powerful tool for the standardization and authentication of HMs.

## Figures and Tables

**Table 1:** List of publications on HMs’ authentication.

No.	Instruments	Chemometrics Techniques	Sample	Brief Results	References
1	HPLC, UPLC, NIR, and CE	PCA and HCA	*Panax notoginseng*	Identification of *Panax notoginseng* can be performed using a combination of analytical chemistry methods and chemometrics techniques.	[33]
2	HPLC	PCA and SIMCA	*Andrographis paniculata* Nees	*A. paniculata* from different origins were successfully classified using SIMCA based on the predefined PCA model.	[51]
3	UPLC-DAD	N/A	*Rosa rugosa*	Authentication of *Rosa rugosa* was carried out and 23 characteristic fingerprint peaks were verified.	[10]
4	HPLC	N/A	*Andrographis paniculata, Cinnamomum zeylanicum, Curcuma xanthorrhiza, Eugenia polyantha,* and *Orthosiphon stamineus*	HPLC fingerprint analysis can be applied for the quality control method for glucofarmaka antidiabetic jamu.	[52,53]
5	LC–MS/MS	N/A	The Sogunjung decoction (Korean traditional medicine)	Eleven marker components in the Sogunjung decoction were detected in amounts of 0.01–51.83 mg/g.	[54]
6	HPLC-PDA and LC–MS/MS	N/A	Hyeonggaeyeongyo-tang (Korean traditional medicine)	The amounts of 20 marker components using HPLC–PDA and LC–MS/MS were determined to be 0.18–14.60 and 0.01–1.76 mg/freeze-dried g, respectively.	[55]
7	RRLC–ESI/QTOF MS	PCA and SVM	*Lonicera* species	Seven *Lonicera* species flower buds were classified with six marker compounds.	[31]
8	^1^H-NMR	PLSA–DA, OPLSA–DA, and PLS	*Curcuma longa, Curcuma heyneana,* and *Curcuma manga*	The authentic *C. longa* samples were successfully separated from the adulterated samples with the good and accurate calibration chemometrics models.	[56,57]
9	^1^H-NMR	PCA and OPLSA–DA	*Curcuma xanthorrhiza* and *Curcuma aeruginosa*	The acceptable discrimination parameters were achieved with a high value of R^2^X, R^2^Y and Q^2^(cum).	[58,59]
10	^1^H-NMR	PCA, PLSA–DA, and N-nearest neighbors (N3)	*Polygoni multiflori Radix,* and *Cynanchi auriculati Radix*	In total, 70.99% data contributions were involved in the PCA model. PLSA–DA and N3 were effective to employ the authentication stage.	[60]
11	^1^H-NMR	PCA and O2PLSA–DA	*Crocus sativus* (Saffron)	Metabolites of pirocrocin (1.12, 1.16, 2.08, 4.28, and 10.04 ppm) and crocins (1.96, 4.16, 5.40, 6.52, 6.64, 6.84, and 7.32 ppm) were found higher in authentic Saffron samples.	[61]
12	^1^H-NMR	PCA and OPLSA–DA	*Capsicum annum* L. (serrano pepper)	Metabolites of citrate, lactate, aspartate, leucine, and sucrose in serrano peppers were successfully identified and classified.	[62]
13	^1^H-NMR	CDA	*Capsicum annuum* L.	Korean, Chinese, and Vietnamese red pepper powders were successfully differentiated.	[63]
14	^1^H-NMR	OPLSA–DA	*Pepper nigrum* L. (black pepper)	PCA was used to differentiate piperine samples from Srilanka, Brazil, and Vietnam. OPLSA–DA model using CD3OD resulted in the value of R2X (0.977), R2Y (0.962), and Q2 (0.928).	[64]
15	^1^H-NMR	PCA and PLSA–DA	*Apium graveolens* L. var. dulce	All samples were correctly classified without misclassification, indicating a good performance of the OPLSA–DA model.	[65]
16	^1^H-NMR	PCA and OPLSA–DA	*Mentha* species	Three species of *Mentha* were classified with good predictive ability (Q2 = 0.978).	[66]
17	^1^H-NMR	PCA and OPLSA–DA	*Cinanmomum verum* and *Cinnamomum cassia*	Eugenol was found to be a potential biomarker of *Cinnamomum verum*, whereas the presence of fatty acids was found as a potential biomarker of *Cinnamomum cassia.*	[67]
18	^1^H-NMR	PCA and OPLSA–DA	*Allium cepa* L.	White onion was characterized by the presence of glucose, sucrose, FOS, and sterols, whereas the red onion contained sterols and glucose. Important metabolites in yellow onion were methiin, free isoalliin, γ-glutamyl-isoalliin, glutamine, malate, and choline.	[68]

## Data Availability

Data available in a publicly accessible repository.
